# The Implementation of the WHO Mental Health Gap Intervention Guide (mhGAP-IG) in Ukraine, Armenia, Georgia and Kyrgyz Republic

**DOI:** 10.3390/ijerph18094391

**Published:** 2021-04-21

**Authors:** Irina Pinchuk, Yulia Yachnik, Oksana Kopchak, Kristine Avetisyan, Khachatur Gasparyan, Gayane Ghazaryan, Eka Chkonia, Lilya Panteleeva, Anthony Guerrero, Norbert Skokauskas

**Affiliations:** 1 Institute of Psychiatry of Taras Shevchenko National University of Kyiv, 03022 Kyiv, Ukraine; 2 Doctor Psychologist, University Clinic of Taras Shevchenko National University of Kyiv, 03022 Kyiv, Ukraine; yuliaya4nik@gmail.com; 3 Department of Neurology, Psychiatry and Physical Rehabilitation, Kyiv Medical University, 02000 Kyiv, Ukraine; dr.kopchak@kmu.edu.ua; 4 Medical Psychology Department, Mkhitar Heratsi Yerevan State Medical University, Yerevan 0025, Armenia; chrisam999@gmail.com (K.A.); kvgaspar@yahoo.com (K.G.); ghazaryan.psy@gmail.com (G.G.); 5 Department of Psychiatry, Tbilisi State Medical University, Tbilisi 0159, Georgia; ekachkonia@gmail.com; 6 Department of Psychiatry, Kyrgyz-Russian Slavic University, Bishkek 720022, Kyrgyzstan; p.lilya12@gmail.com; 7 Child and Adolescent Psychiatry Division, University of Hawai’i John A. Burns School of Medicine, Honolulu, HI 96813, USA; GuerreroA@dop.hawaii.edu; 8 Centre for Child and Adolescent Mental Health and Child Protection, Institute of Psychiatry, Trondheim Norwegian University of Science and Technology, 7491 Trondheim, Norway; norbert.skokauskas@ntnu.no

**Keywords:** GAP, mental health, implementation, Intervention Guide

## Abstract

Despite the increasing burden of mental disorders, a lot of people worldwide suffer a gap in receiving necessary care in these countries. To close this gap, the WHO has developed mhGAP training modules aimed at scaling up mental health and substance use disorders services, especially in low- and middle-income countries. This article presents the experience of implementing the Mental Health Gap Action Programme (mhGAP) in Ukraine, Armenia, Georgia, and Kyrgyz Republic. Data were gathered from an electronic questionnaire administered to representatives from higher educational institutions where the Mental Health Gap Action Programme Intervention Guide (mhGAP-IG) was implemented in existing curricula for medical students, interns, and residents in family medicine and neurology, practicing physicians, and master’s program in mental health students. More than 700 students went through the programs that provided the feedback. Evaluations of program effectiveness mainly involved standard discipline tests or pre- and post-tests proposed in the mhGAP trainer manual. This finding suggested that mhGAP-IG can be successfully adapted and implemented both on undergraduate and on postgraduate education levels and among medical and nonmedical specialists. Future evaluations need to more definitively assess the clinical effectiveness of mhGAP-IG implementation.

## 1. Introduction

Mental and behavioral disorders are highly prevalent across the globe. The World Health Organization (WHO) states that one in four people could have a mental health disorder during their lifetime [1]. In 2016, about 16% of the world’s population was affected by a mental or behavioral disorder, which caused 162.5 million DALYs (disability-adjusted life years) loss [2]. About two-thirds of the mental health burden is caused by depressive, anxiety, drug use, and alcohol use disorders [2]. Despite the high prevalence of mental, neurological, and substance use disorders, the significant treatment gap continues to exist in many countries [3,4,5]. More than 60% of countries spend 1% or less of their budget on mental health. Mental health workers account for only 1% of the global health workforce, and 40% of countries have less than one mental health hospital bed per 10,000 people [1].

While mental and behavior disorders increase risk for noncommunicable diseases, and injuries [6,7], individuals with mental disorders rarely seek help, and poor adherence to treatment can negatively impact their prognosis [8]. Stigma can be a major factor preventing people with mental health disorders from seeking help [9]. Thus, countries, particularly low- and middle-income countries (LMIC), need to scale up mental health services coverage.

Ukraine, Armenia, Georgia, and Kyrgyz Republic are former Soviet republics, which have walked down different pathways after gaining independence in 1991.

Ukraine is located in Eastern Europe and is the second largest country in the European region. It has a population of 42.0 million [10]. After gaining independence, Ukraine experienced several large political changes, including the “Orange Revolution” in 2004 and the “Revolution of Dignity” in 2014 [11]. Recent military conflict, started in 2014, has led to a humanitarian crisis with about 3.4 million people in need of humanitarian assistance, and about 1.4 million people internally displaced [12].

Georgia is a country in the Caucasus region located at the crossroads of Western Asia and Eastern Europe. The population of Georgia is 3.7 million [13]. Since independence, Georgia went through different crises, including secessionist conflicts in Abkhazia and South Ossetia, the Rose Revolution in 2003, and the Russo-Georgian War in August 2008 [14]. Nevertheless, economic growth during the last years has been robust and resilient, resulting in significant reduction of the poverty rate [13].

Armenia is located in the Western Asia, South Caucasus region. The population of Armenia is 2.9 million [15]. The initial years of independence were marked by economic difficulties, rooted in the Karabakh conflict with Azerbaijan (1988–1994). In 2018, Armenia passed through political changes due to the “Velvet Revolution”. In the same year, Armenia became an upper-middle-income country with its gross national income per capita [16].

The Kyrgyz Republic is a lower-middle-income country of 6.5 million people in Central Asia. Since independence in 1991, the country has experienced instability, with political and social upheavals in 2005 and 2010 [17].

Additional information on the countries can be found in Table 1.

Development of mental health systems in the Eastern Europe and the Asian region is characterized by gradual decentralization, with often disproportionately slow growth of community services [18]. In many countries, the development of services was influenced by humanitarian crises and disasters, dictating the need to adapt and meet specific catastrophe-related needs. The philosophy of the biological model of mental disorders weakened, and attention to the social and psychological aspects increased across the region. However, development, to a large extent, is supported by international organizations, rather than by systematic implementation of government policies [19,20].

The estimated prevalence and burden of mental health and substance use disorders in Ukraine, Georgia, Armenia, and Kyrgyz Republic are shown in Table 2. Data show that, in Ukraine, there is a higher prevalence of depression, anxiety, and alcohol use disorder compared with other countries, and a higher prevalence of depression and alcohol use disorder compared with globally. Georgia and Armenia have similar prevalence rates, but in Armenia the mental health burden is higher. In Kyrgyz Republic, the prevalence of mental health disorders is lower than in other comparison countries, with relatively high shares of alcohol use disorder.

Mental health gaps remain high in the region [3,4]. The recent assessment of mental health services in Ukraine in 2019 showed that most people (up to 75%) with common mental disorders and alcohol use disorder have limited access to adequate mental health services [21]. The existing mental health services in Armenia are mostly centralized, with low representation at the outpatient or community level [22], and do not reflect the burden and actual need [16,23]. Mental health services in Kyrgyz Republic experience insufficient financing, lack of equipment, underregulation, a deficiency of qualified specialists, and, as a result, suboptimal quality of mental health care [24,25,26]. Stigmatization is still a substantial problem in these countries, as it prevents people from receiving appropriate care and compounds the challenges associated with lack of awareness, low quality, and insufficient geographical and financial accessibility of services [21,22,27].

Incorporation of mental health into specialized and primary care services could be a possible solution to existing challenges and could reduce the burden associated with mental disorders [28,29,30,31]. Indeed, given the low numbers of psychiatrists relative to the general population, as indicated in Table 1, it is unlikely that psychiatrists alone can sufficiently meet the population’s mental healthcare needs, and primary care providers and other healthcare professionals will need to be significantly involved in delivering competent and accessible mental healthcare. However, training of medical doctors on mental health problems is not always sufficient. Thus, in Armenia, only 5% of the training for medical doctors is devoted to mental health. The training is even less for nurses and comprises only 1% of their undergraduate curriculum [22]. In Kyrgyz Republic, 4% of the medical doctor training program is devoted to mental health in comparison to 5% of the program for nurses [24]. General practitioners in Ukraine perceive that time spent on mental health at the university was not enough and express great interest in receiving additional mental health training [21]. In most countries of the East European WPA (World Psychiatric Association) zone, the unified system of continuous postgraduate education for general practitioners in mental health is absent [32]. Training programs for psychiatrists may need improvement as well [32,33].

In order to scale up mental health and substance use disorders services, WHO, in 2008, launched the Mental Health Gap Action Programme (mhGAP). To assist in implementation of mhGAP, WHO developed the Mental Health Gap Action Programme Intervention Guide (mhGAP-IG), which provides evidence-based guidelines, tools, and clinical decision-making protocols for integrated management of priority conditions. The mhGAP-IG has been developed for use by healthcare providers working in nonspecialized healthcare settings, with adaptation to meet national and local needs [5,34].

According to a recent systematic review by Keynejad and colleagues, the mhGAP-IG was successfully used by clinicians, government ministries, trainers, educators, and academics in a range of LMICs [5]. Forms and modules used vary based on local adaptation, training, and clinical practice, and include mobile applications, tablet-based avatar-assisted family training, and novel rating scales. However, the review above did not include studies from Ukraine, Armenia, Georgia, or Kyrgyz Republic, and outlined only one study about the use of the mhGAP-IG in a higher education setting.

There were several initiatives in Ukraine to implement the mhGAP program. In 2017, at the request of the Ministry of Health of Ukraine, WHO supported the launch of capacity-building activities for the mhGAP Humanitarian Intervention Guide in Eastern Ukraine. In 2019, it expanded the program and trained primary healthcare workers on Version 2.0 of the mhGAP Intervention Guide in the Donetsk region [35]. However, those initiatives so far remain limited in certain regions and, because they are not funded by government, may lack sustainability.

At the same time, ongoing and sustainable development of a mental health workforce should include strengthening of institutional capacity to implement training programs effectively [34]. One of the possible solutions to bridge the gap in the field of mental health care and to ensure stable and long-term changes is the implementation of mhGAP-IG into existing pre- and postgraduate training curricula [36,37]. Integration of mhGAP-IG into pre-service training could bring long-term benefits, including sustainability, cost-effectiveness, opportunity to develop a shared view among different mental health specialists, long-term strengthening of health systems through building the workforce’s capacity to provide high-quality, evidence-based care, and having a reference point for later in-service training [36]. Therefore, integration of mhGAP-IG into education settings is a promising and important public health opportunity that deserves further study.

Ukraine, Armenia, Georgia, and Kyrgyz Republic work together towards strengthening mental health education within the framework of the Ukraine-Norway-Armenia (UNA) Partnership. The UNA Partnership initially included higher education institutions (HEIs) from Ukraine, Norway, and Armenia. Later, UNA Partnership activities were joined by Tbilisi State Medical University (Georgia) and Kyrgyz-Russian Slavic University (Kyrgyz Republic). Among other initiatives, the UNA Partnership focused on mhGAP-IG implementation in selected HEIs.

The initiative of WHO mhGAP-IG implementation in pre-service training was discussed and developed during three consultative meetings in 2018, which took place concurrently with the 23rd World Congress of the International Association for Child and Adolescent Psychiatry and Allied Professions, the 18th WPA World Congress of Psychiatry, and the 2018 mhGAP Forum held at WHO’s headquarters in Geneva, Switzerland.

Later in 2018, in Kyiv, Ukraine, a three-day introductory mhGAP course brought together decision makers and clinical educators from postgraduate and undergraduate teaching institutions to discuss how mhGAP-IG can be used to strengthen pre-service training and to provide training on mhGAP-IG, with special emphasis on the module on child and adolescent mental and behavioral disorders. There were 20 faculty members from medical universities in Ukraine, Georgia, Armenia, and Kyrgyz Republic. Each University went through a preparation and adaptation phase, when specific modules and required number of hours were decided based on the existing curriculum and needs [36].

This article provides a one-year follow-up on the implementation of mhGAP-IG in educational settings in Ukraine, Armenia, Georgia, and Kyrgyz Republic after initial assessment [36].

The aim of this study was to collect one-year follow-up data on implementation of mhGAP-IG in education settings in Ukraine, Armenia, Georgia, and Kyrgyz Republic. The objectives were to collect information about curriculum changes of existing courses, development of new ones, teaching processes, and number of students one year after mhGAP-IG implementation; to collect information about evaluation methods used to assess effectiveness of mhGAP-IG implementation in education settings, and to gather recommendations from faculty members who have implemented the mhGAP-IG. We believe that information on the practical realities of implementing standardized training modules in different world regions is essential in optimally planning how to prepare the diverse global workforce of healthcare providers who have the potential to address a critically important healthcare need.

## 2. Materials and Methods

### 2.1. Study Design

A cross-sectional descriptive study was conducted from October 2019 to February 2020. Data were gathered from an electronic questionnaire.

### 2.2. Study Sample

Participants were at HEIs in Ukraine (Kyiv Medical University, Department of Neurology, Psychiatry and Physical Rehabilitation), Armenia (Yerevan State Medical University, Department of Medical Psychology), Georgia (Tbilisi State Medical University and Ilia State University), and Kyrgyz Republic (Kyrgyz-Russian Slavic University and Kyrgyz State Medical Institute of retraining and advanced training).

### 2.3. Data Collection Tool

For the purpose of the study, a self-administered electronic questionnaire was developed. The questionnaire consisted of 20 questions, both open- and closed-ended, divided into four sections: (a) background (i.e., information about the respondent and institution); (b) current use of mhGAP-IG in education settings (curriculum development/modification, number of hours, modules used, teaching process, number of students trained, etc.); (c) evaluation of effectiveness of mhGAP-IG implementation in education settings (methods used, data available, etc.); (d) recommendations (identified strengths, challenges, etc.).

### 2.4. Data Analysis

Descriptive data are presented as case examples and in tabulated form.

### 2.5. Ethics Approval and Consent to Participate

Implicit consent was provided when respondents completed the questionnaire.

## 3. Results

Cumulative results are presented in Table 3.

### 3.1. Case Example: Ukraine

*Background:* The official WHO Ukrainian translation of mhGAP-IG has been available since 2019. Kyiv Medical University implemented the mhGAP-IG-enhanced curriculum in September 2019. The existing training curriculum on Psychiatry and Narcology for 4th-year medical students was revised in several steps to implement mhGAP-IG. The mhGAP-IG was presented to the staff of the department of Neurology, Psychiatry and Physical Rehabilitation of Kyiv Medical University, and possible changes in the curriculum were discussed. Necessary changes have been introduced, and the updated curriculum on Psychiatry and Narcology was approved by the Scientific Council in June 2019 and signed by vice-rector of the Kyiv Medical University in August 2019. Additionally, in 2019, following the example of undergraduate curriculum, the curriculum for Neurology residents was updated using mhGAP-IG.

#### 3.1.1. Use of mhGAP-IG in Training

The following modules were selected: dementia, epilepsy, substance use disorders, and child and adolescent mental and behavioral disorders. The introduction of the mhGAP-IG allowed reduction of didactic sessions while maintaining the same number of hours spent on interactive practical sessions and emphasizing self-study of mhGAP-IG online materials [38]. The resources used in education included videos, role play scenarios, and review of the mhGAP-IG printed version and presentations and handouts created by faculty. The modules were taught by academic clinicians using a mixture of didactic teaching, group activities, and clinical tools during residency at Kyiv Medical University—trained 4th-year medical students and medical residents in neurology using mhGAP-IG modules.

The mhGAP-IG has been received very well by the students, clinical instructors, and clinical administrators. Identified benefits include improved trainee knowledge about mental disorders.

#### 3.1.2. Evaluation

The program was evaluated based on students’ and residents’ performance (clinical case discussions in the classroom, tests, and exams), which showed improved knowledge, compared to in the standard curriculum, about mental disorders. Additionally, faculty members discussed their perceptions on the updated program at Scientific Council and expressed positive attitudes towards the mhGAP-IG.

The students and residents were given the survey to assess their level of involvement and feedback about strengths and weaknesses of the modules of mhGAP-IG implemented in the program. In their feedback, both students and residents reported acquisition of better knowledge and skills to identify and manage mental disorders. On the other hand, teachers using different assessment measures (tests, clinical tasks, and cases discussion) found improvement of the students results on the topics after implementation of the mhGAP-IG modules concerning their ability to recognize the symptoms, to put the correct diagnosis, and to provide appropriate treatment of mental diseases.

#### 3.1.3. Outcomes

The implementation of mhGAP-IG was effective at both undergraduate and postgraduate levels of medical education. Well-structured information and visual materials have been identified as the main advantages of the mhGAP-IG training and implementation process. The program was evaluated based on students’ and residents’ performance and feedback surveys.

### 3.2. Case Example: Armenia

#### 3.2.1. Background

The working group of the department initiated the translation of the textbook into Armenian. Staff members of Yerevan State Medical University, Department of Medical Psychology, developed and implemented programs for both undergraduate and postgraduate training levels. Adaptation of the mhGAP-IG-based curriculum took approximately 1 month.

#### 3.2.2. Use of mhGAP-IG in Training

The pre-service curriculum for the second-year undergraduate students was updated with mhGAP and included eight academic hours for child and adolescent mental and behavioral disorders (with emphasis on attention deficit and hyperactivity disorder), depression, and suicide. Two modules of a continuing medical education (CME) for senior and mid-level medical professionals have also been developed as two-week, 60-h courses entitled “Mental Health of Children and Adolescents” and “Mental Health of Primary Care for Children and Adolescents.” The programs were attended by 11 pediatricians and 30 specialists (psychologists, social workers, social educators, and legal advisers) from 11 orphanages and social services centers for the elderly and disabled. Additionally, a CME course on psychological aspects of palliative care was adapted using mhGAP-IG and attended by 27 oncologists. Additionally, it is planned to develop a CME course in conjunction with the Yerevan State Medical University Department of Family Medicine for doctors and nurses in the primary care setting. Three topics from mhGAP were also included in the curriculum of Psychology courses at American University of Armenia. Furthermore, several mhGAP topics were included as part of the training curriculum of military psychologists from the Ministry of Defense of the Republic of Armenia.

#### 3.2.3. Evaluation

The program was evaluated based on students’ and trainees’ performance (tests). Additionally, feedback questionnaires for 2-year students were used and showed that more than 85% of trainees found the course to be very useful [39].

#### 3.2.4. Outcomes

The program was evaluated based on students’ and residents’ performance and feedback surveys. Feedback from participants showed that mhGAP-IG pretraining can be successfully implemented in various conditions with different students (doctors and nurses) and can be adapted to meet the unique needs of each institution. It can be assumed that the introduction of mhGAP-IG techniques and approaches is possible and acceptable, with adequate preparation and under certain conditions.

### 3.3. Case Example: Georgia

#### 3.3.1. Background

In 2018, Georgia joined the UNA (Ukraine–Norway–Armenia) partnership project. mhGAP-IG has been translated into the Georgian language and included into the pre-services curriculum and accredited CME courses for medical doctors of different specialties. The initial adaptation into the pre-service curriculum took around 1 week.

#### 3.3.2. Use of mhGAP-IG in Training

The mhGAP-IG has been integrated into the medical student curriculum at Tbilisi State Medical University and into the Medical Academy curriculum for fourth- and fifth-year students. Accredited CME courses for primary healthcare professionals have been prepared. The mhGAP-IG has been integrated into the psychiatry clerkship and psychiatry internship syllabus. The chapters on psychoses, depression, dementia, self-harm/suicide, and child and adolescent mental and behavioral disorders were added to the curriculum of the mental health master’s program at Ilia State University. In general, the learning process using the WHO mhGAP-IG was highly interactive. All students had the opportunity to actively participate in the discussion and role play process, which they liked very much.

#### 3.3.3. Evaluation

For knowledge assessment, the questionnaires from the mhGAP module for psychoses and child and adolescent mental health were used only as post-tests. The comparison of pre- and post-tests was not conducted. The average score on post-test was 82%. After each semester, the university usually assesses the satisfaction of students with the course. This year, the highest marks were received, even though only online classes were provided. Students stated that the WHO mhGAP-IG is a well-structured tool with practical mhGAP-IG supplementary materials that helped them to understand and realize the difficulties and consequences of mental health problems. The personal stories gave them clear messages regarding patients’ and families’ challenging experiences. The video instructions were very helpful in terms of learning interviewing skills, which the students could practice with the role-playing exercises. At the same time, students pointed out that they lacked sessions with real patients.

#### 3.3.4. Outcomes

mhGAP-IG was successfully incorporated on different levels of education, including in specialized mental health courses. Well-developed manuals and supporting materials make mhGAP-IG easy to implement. Support from the university leadership is necessary during the implementation process. The program was evaluated based on students’ and residents’ performance (using mhGAP test in the manual) and feedback surveys.

### 3.4. Case Example: Kyrgyz Republic

#### 3.4.1. Background

The mhGAP guideline was translated into the Kyrgyz language. A seminar on adaptation and implementation of mhGAP was conducted. Order No. 749 of the Ministry of Health of the Kyrgyz Republic of 7 July 2019 was signed, approving the usage of mhGAP in the training of medical personnel in universities of the country at the graduate and postgraduate levels. At the first stage, 20 trainers were trained to further teach the mhGAP program. Then, in the capital of the republic, a pilot mhGAP training was conducted, in which 15 general practitioners, 10 psychologists, and 15 nurses providing outpatient mental healthcare from 10 pilot regions participated. The training was evaluated and recommended for implementation in education settings. With support from the Ministry of Health, mhGAP was implemented in the educational sector at the graduate and postgraguate levels.

Before the approval of the program by the Government of the Kyrgyz Republic for the protection of mental health of the population for 2018–2030 and the introduction of mhGAP in the country at the undergraduate and postgraduate levels, training in the subject of psychiatry only involved study of the following: the main criteria of the ICD-10; the main psychopathological symptoms such as disturbances in perception, thinking, emotions, motor function, and consciousness; psychopharmacology. After Government approval of and implementation of the mhGAP, the emphasis in teaching psychiatry shifted to practical management of the patient and provision of comprehensive care, including psychoeducation, stress management, and psychosocial care. In the final exam for medical students, questions related to mental health or to the study of behavioral sciences are almost not included. This absence creates a steady depreciation of psychiatric or psycho-educational skills among medical students. In Kyrgyzstan there is no system to reward general practitioners for identifying and treating patients with mental disorders. Recently, a program of monetary incentives for doctors has been developed; however, it is currently at the stage of pilot testing.

#### 3.4.2. Use of mhGAP-IG in Training

Four topics were selected to implement the mhGAP guidelines in Kyrgyzstan: depression, suicide/self-harm, childhood and adolescent mental and behavioral disorders, and substance abuse. mhGAP topics have been included in the curriculum of medical institutes and universities (Kyrgyz-Russian Slavic University—18 h and Kyrgyz State Medical Institute of retraining and advanced training—37 h). The Department of Family Medicine of the Kyrgyz State Medical Institute of retraining and advanced training provides postgraduate training for family doctors and nurses.

#### 3.4.3. Evaluation

The evaluation is based on post-training feedback questionnaires as well as pre- and post-tests from mhGAP. Evaluation of the training showed that 75% of general practitioners rated mhGAP guidelines as relevant and necessary for their practice. In addition, training was also evaluated based on medical records. Detection of depression and suicide among the population from regions where practitioners participated in the pilot training increased by 17%. Based on these results, it was recommended to include mhGAP-IG in the curriculum of medical HEIs.

#### 3.4.4. Outcomes

The mhGAP-IG was successfully implemented on under- and postgraduate levels. The program was evaluated based on students’ and residents’ performance (using mhGAP test in the manual) and feedback surveys. Additionally, efficacy of practitioners training was evaluated based on medical records. Implementation of the mhGAP-IG contributed to increased awareness about mental health and recognition of mental health professionals.

## 4. Discussion

The mhGAP-IG has been successfully implemented in all four countries at both undergraduate and postgraduate levels. In all cases, the importance of support and recognition by the administration was pointed out. In one case (Kyrgyzstan), legislative changes and support were needed to permit the introduction of mhGAP-IG in educational programs at the country level.

At the undergraduate level, the program was introduced into existing curricula for students of different years of study, including second year (Armenia), fourth year (Ukraine), and sub-internship year (Georgia). At the postgraduate level, existing programs were revised or new ones created based on mhGAP-IG. In two countries (Armenia and Kyrgyzstan), mhGAP-IG has been included in postgraduate programs for general practitioners. In Ukraine and Armenia, postgraduate programs for doctors of other specialties (resident neurologists, oncologists, and pediatricians) were developed based on or using mhGAP-IG. In one country (Armenia), a postgraduate educational program for nonmedical specialists (psychologists, social workers, social educators, and legal advisers from orphanages and social centers for children) was developed based on mhGAP-IG. Only in one country (Kyrgyzstan), educational programs including mhGAP-IG targeted for both family doctors and nurses. In one country (Georgia), mhGAP-IG modules were included in the educational program of psychiatry specialists (specialization in Psychiatry and Social Psychiatry Master’s program).

All countries implemented modules on depression, suicide, and child and adolescent mental and behavioral disorders. Three countries implemented the module on substance use disorders, and two countries (Ukraine, Georgia) implemented modules on dementia. Only Ukraine implemented the module on epilepsy. Only Georgia implemented the modules on psychosis and other significant mental health complaints.

The scope and nature of evaluating the effectiveness of introducing mhGAP-IG into educational programs varied from country to country (Table 4), but mainly it was performed using standard discipline tests or pre- and post-tests proposed in the mhGAP trainer manual. In Georgia, the feedback questionnaires examining student satisfaction at the end of each course were used. In one case (Kyrgyzstan), pilot in-service training was evaluated based on a change in knowledge and indicators from medical records. Based on this assessment, the further implementation of mhGAP-IG in educational programs was recommended.

### Strengths and Limitations of the Study

The study is limited in its inclusion of only four countries, united by similarities in socioeconomic background and implementation of mhGAP-IG during a similar timeframe. It is envisioned that this description of the process of implementing mhGAP-IG in these countries can help other countries that may be interested in introducing what appears to be a helpful tool in advancing the population’s access to mental healthcare. There is also the possibility of self-reporting bias, as the questionnaire were self-administered.

## 5. Conclusions

This study shows that the mhGAP-IG can be successfully adapted and implemented on both undergraduate and postgraduate education levels for medical and allied specialties.

## Figures and Tables

**Table 1:** Ukraine, Georgia, Armenia, and Kyrgyz Republic: countries context.

Indicator	Ukraine	Georgia	Armenia	Kyrgyz Republic
Population	42.0 ^2^	3.7 ^2^	2.9 ^2^	6.5 ^2^
Region	Eastern Europe	Western Asia	Western Asia	Central Asia
Income group (WB 2019 classification)	Lower-middle income ^2^	Upper-middle income ^2^	Upper-middle income ^2^	Lower-middle income ^2^
GDP, current $ billion	153.2 ^2^	15.7 ^2^	12.4 ^2^	8.5 ^2^
GDP per capita, current US$	3649 ^2^	4764 ^2^	4643 ^2^	1323 ^2^
Life expectancy at birth (years), 2018	72 ^3^	74 ^3^	75 ^3^	71 ^3^
Current health expenditures	177 ^3^	293 ^3^	408 ^3^	79 ^3^
Burden of mental disorders (DALYs per 100,000 population)	3416.26 ^1^	3508.45 ^1^	3266.01 ^1^	1273.51 ^5^
Psychiatrists per 100,000 population	6.93 ^1^	6.71 ^1^	3.84 ^1^	3.51 ^4^
Child psychiatrists per 100,000 population	0.76 ^1^	0.30 ^1^	0.27 ^1^	n/a
Total mental health workers per 100,000	8.95 ^1^	9.34 ^1^	27.39 ^1^	27.5 ^4^
Mental hospital beds per 100,000	65.48 ^1^	36.72 ^1^	n/a	31.74 ^4^
General hospital psychiatric unit beds per 100,000	1.92 ^1^	2.53 ^1^	1.71 ^1^	1.75 ^4^

Sources: ^1^ WHO Mental Health Atlas 2017. ^2^ https://data.worldbank.org/ (accessed on 16 October 2020). ^3^ World Health Organization Global Health Expenditure database. ^4^ WHO-AIMS Report on Mental Health System in Kyrgyzstan, WHO and Ministry of Health, Bishkek, Kyrgyz Republic, 2008. ^5^ Global Burden of Disease 2017 http://ghdx.healthdata.org/gbd-results-tool (accessed on 16 October 2020). Abbreviations: DALYs—disability-adjusted life years. GDP—gross domestic product. WB—The World bank.

**Table 2:** Estimated prevalence and burden of mental health and substance use disorders in Ukraine, Georgia, Armenia, and Kyrgyz Republic.

Indicator	Ukraine	Georgia	Armenia	Kyrgyz Republic	LMIC	Global
Mental disorders prevalence, %	13.7	11.28	11.19	10.69	12.97	13.17
DALY, %	3.63	3.58	4.59	4.77	4.06	4.89
Schizophrenia prevalence, %	0.24	0.23	0.23	0.18	0.21	0.27
DALY, %	0.30	0.36	0.47	0.40	0.35	0.51
Depressive disorders prevalence, %	5.18	3.36	3.19	2.79	3.27	3.59
DALY, %	1.71	1.30	1.59	1.65	1.42	1.72
Anxiety disorders prevalence, %	3.18	2.78	2.76	2.47	3.54	3.86
DALY, %	0.60	0.65	0.83	0.85	0.87	1.08
Alcohol use disorder prevalence, %	5.47	2.21	2.17	2.71	1.22	1.46
DALY, %	2.65	0.71	0.9	1.87	0.51	0.70
Drug use disorder prevalence, %	0.70	0.48	0.52	0.54	0.74	0.97
DALY, %	0.75	0.48	0.57	0.78	0.69	1.09

Source: Global Burden of Disease 2017. http://ghdx.healthdata.org/gbd-results-tool (accessed on 16 October 2020). Abbreviations: DALY—disability-adjusted life year. LMIC: low- and middle-income countries.

**Table 3:** Implementation of the Mental Health Gap Action Programme Intervention Guide (mhGAP-IG) into education settings in Ukraine, Armenia, Georgia, and Kyrgyz Republic.

Country	University	Education Level for mhGAP Implementation	Selected mhGAP Modules	Target Audience	Number of Hours	Number of Students Trained
Ukraine	Kyiv Medical University, Department of Neurology, Psychiatry and Physical Rehabilitation	Undergraduate	Dementia Epilepsy Substance use disorders Child and adolescent mental and behavioral disorders	Medical students of 4th year of study	10	93
		Postgraduate	Dementia Epilepsy Substance use disorders Child and adolescent mental and behavioral disorders	Neurological residents	10	n/a *
Armenia	Yerevan State Medical University, Department of Medical Psychology	Undergraduate	Child and adolescent mental and behavioral disorders Depression Suicide	Medical students of 2nd year of study	8	520
		Postgraduate	Child and adolescent mental and behavioral disorders Depression Suicide	Family doctors (CME)	12	51
			Child and adolescent mental and behavioral disorders	Pediatricians (CME)	60	12
			n/a *	Oncologists (CME)	n/a *	27
			Child and adolescent mental and behavioral disorders Depression Suicide	Family Medicine 1 and 2 year residents	12	14
			Child and adolescent mental and behavioral disorders	Psychologists, social workers, social educators, legal advisers from 11 orphanages and social centers	60	30
Georgia	Tbilisi State Medical	Undergraduate (clerkship)	Child and adolescent mental and behavioral disorders Dementia Suicide Other significant mental health complaints	Medical students 8 semester	10	41
		Sub internship	Child and adolescent mental and behavioral disorders Substance use disorders	Medical students 10 semester	8	32
		MD Psychiatry	Child and adolescent mental and behavioral disorders Psychosis Depression	Medical students 9, 10 semester	8	116
	Ilia State University	Social Psychiatry Master program	Child and adolescent mental and behavioral disorders Psychosis Depression Dementia Suicide and other significant mental health complaints Substance use disorders	Master students 2 semester	14	5
Kyrgyz Republic	Kyrgyz-Russian Slavic University	Undergraduate	Depression Suicide Substance use disorders Child and adolescent mental and behavioral disorders	Medical students	18	96
	Kyrgyz State Medical Institute of retraining and advanced training	Postgraduate	Depression Suicide Substance use disorders Child and adolescent mental and behavioral disorders	Family doctors	37	160
				Nurses		35

* Data was not provided.

**Table 4:** Evaluation of mhGAP-IG implementation.

Evaluation Method	Ukraine	Armenia	Georgia	Kyrgyz Republic
Students’ performance according to standard tests and exams	+	+		
Pre- and post-tests from mhGAP-IG			+	+
Feedback survey of students		+	+	+
Feedback survey of faculty members	+			
Medical records (number of mental health diagnosis) before and after training of medical doctors				+
+				

## Data Availability

The data presented in this study are available on request from the corresponding author.
